# Carbohydrates in Cyberspace

**DOI:** 10.3389/fimmu.2015.00300

**Published:** 2015-06-10

**Authors:** Elizabeth Yuriev, Paul A. Ramsland

**Affiliations:** ^1^Monash Institute of Pharmaceutical Sciences, Monash University, Parkville, VIC, Australia; ^2^Centre for Biomedical Research, Burnet Institute, Melbourne, VIC, Australia; ^3^Department of Immunology, Alfred Medical Research and Education Precinct, Monash University, Melbourne, VIC, Australia; ^4^Department of Surgery Austin Health, University of Melbourne, Heidelberg, VIC, Australia; ^5^CHIRI Biosciences, School of Biomedical Sciences, Curtin University, Perth, WA, Australia

**Keywords:** Carbohydrate, curation, glycan, molecular modeling, online database, three-dimensional structure

Many research areas, particularly those focused on structural aspects of biomolecules, have “moved into cyberspace” in the last 20–30 years. This move has become even more prominent in the last 5–10 years, amplified by the increased space accessibility (from mobile devices to the Cloud) and by ever more sophisticated and powerful resources available for high-performance computing. The field of structural glycobiology has greatly benefitted from such progress and has taken advantage of the computational tools and resources developed specifically for the structural analysis of carbohydrates. In this short opinion piece, we do not intend to provide a comprehensive coverage of all such resources. We will briefly survey four websites [GLYCAM, UniCarb KnowledgeBase (UniCarbKB), Glycosciences.de, and Glyco3D], offering tools for structural glycobiology, and will provide some examples of studies where such tools have been successfully used for research relevant to understanding of carbohydrate recognition by proteins involved in immunity and infection. For a more comprehensive listing of computational resources for structural glycobiology, readers should consult Ref. (1–4).

GLYCAM-Web (5) is focused on the prediction of three-dimensional (3D) structures of carbohydrates and macromolecular structures that include carbohydrates. The server is created and operated by the research group of Prof. Robert J. Woods in the Complex Carbohydrate Research Center (CCRC) (6) at the University of Georgia in Athens. The server can perform conformational modeling of oligosaccharides as well as 3D modeling of glycoproteins. The main interfaces for modeling oligosaccharide conformations are the Carbohydrate Builder [including a special Builder for glycosaminoglycans (GAGs)], the Glycoprotein Builder, and the Oligosaccharide Libraries. The server is user-friendly and allows a range of upload and download options. Given a range of file formats – often, program-specific – used in molecular modeling, user-friendliness related to the portability of file formats is a significant “real-estate” feature highly valued by the cyberspace dwellers.

Most of the back-end software used by the server is open source and freely available to the public. This feature is also highly valued by viewers. The reason is not only reduced cost but also the openness of science that could be performed with open-source software. We consider such open access as critical in the field of molecular modeling: it allows researchers to use the program codes and develop them further, thus advancing the development of better modeling methods.

The main engine room of the server is the GLYCAM force field, the outcome of more than 20 years of development. Its latest incarnation, GLYCAM06 (7), has been significantly modified to satisfy the following requirements: transferability of the parameter set to all carbohydrate ring conformations and sizes, carbohydrate derivatives, and other biomolecules; self-containment and transferability to many quadratic force fields; ability to treat both α- and β-anomers without specific atom types. The GLYCAM-Web server (5) is a very widely and extensively used resource. Its statistics records show that an average of 1700–1800 unique users access the site monthly (data examined: December 2014–March 2015).

The GLYCAM-Web server (5) and the GLYCAM force field (7) were used for the investigation of recognition specificity of ABO blood group antigens by antibodies (8). Such circulating antibodies could cause a hyperacute immune response and sometimes death resulting from mismatched blood transfusion or organ transplantation (9). Another recent example of application of GLYCAM was in the study of structure and immune recognition of HIV-1 envelope (10), where it was used for modeling the *N*-linked glycan shield.

UniCarb KnowledgeBase (11, 12) was conceived to support online data storage and search capabilities for glycomics and glycobiology by integrating structural, experimental, and functional information. The aim of UniCarbKB is to advance the understanding of structures, pathways, and networks involved in glycosylation and processes mediated by carbohydrates. This information-rich resource is freely accessible, supports data annotation, and promotes adoption of common standards, necessary for seamless and meaningful integration of structural and functional data. UniCarbKB is the outcome of the collaborative effort of researchers from Australia (Macquarie University and University of NSW), Germany (Max Planck Institute of Colloids and Interfaces), Ireland (National Institute for Bioprocessing Research and Training), Japan (Soka University), Sweden (University of Gothenburg), Switzerland (Swiss Institute for Bioinformatics and University of Geneva), and USA (CCRC).

UniCarb KnowledgeBase provides querying interfaces for three-structural databases (GlycoSuiteDB, EUROCarbDB, and GlycoBase) and a link to the UniCarb-DB (13). UniCarbKB continues efforts started with GlycoSuiteDB (14) in curating structural glycobiology information from research literature. GlycoBase (15) and UniCarb-DB (16) are databases of experimental glycan structures. Support for EUROCarbDB (17) is planned for the future release.

At the time of writing, over 906 references, 3238 glycan structure entries, and 898 glycoproteins have been curated in UniCarbKB. A total of 598 protein glycosylation sites have been annotated using experimentally confirmed glycan structures. Beyond storing, curating, and providing searchable access to structural and functional data, UniCarbKB offers a range of tools for data pre-process and analysis: GlycanBuilder (18, 19) – for fast and intuitive drawing of glycan structures; GlycoMod tool (20, 21) – for predicting the possible glycan structures on proteins, from their experimentally determined masses. UniCarbKB is connected to the GLYCAM site (described above) and RINGS – a web resource offering algorithms and data mining tools for glycobiology research (22). Further, through its SugarBindDB module, UniCarbKB is linked to the Functional Glycomics Gateway of the Consortium for Functional Glycomics (CFG) (23) and the Glyco3D Portal for Structural Glycosciences (24, 25).

The GlycoMod tool of the UniCarbKB was used in a study of the formation of subcellular-specific *N*-glycosylation glycoprotein determinants (26). Lee et al. used liquid chromatography and mass spectrometry-based quantitative glycomics to investigate eight human breast epithelial cells with diverse genotypes and phenotypes – mostly human breast cancer cell lines – and have shown that the secreted glycoproteins consistently displayed more processed, primarily complex type, *N*-glycans than the high-mannose-rich microsomal glycoproteins. They have also demonstrated that secreted glycoproteins displayed significantly more α-sialylation and α-1,6-fucosylation, but less α-mannosylation, than both cell-surface and microsomal glycoproteomes.

Of particular interest to those working on the glycobiology of infectious disease is the SugarBindDB module of UniCarbKB – a database of pathogen lectins and corresponding glycan targets (27, 28). The SugarBindDB advances a pathogen-capture technology specifically for the binding of pathogen lectins to carbohydrate epitopes. The contents of the database come from publicly accessible information. After experts (glycobiologists, microbiologists, and medical histologists) locate candidate papers, the binding data is extracted for human pathogens and specific glycans.

The SugarBindDB database can be searched using names of different entities involved in the carbohydrate-protein interactions. For pathogenic agents (e.g., *Pseudomonas aeruginosa*), the outcomes include agent type and taxonomy ID. For lectins themselves (e.g., PA-IIL), the database stores genes, Protein Data Bank (PDB) codes, and associated Glyco3D and CFG references (see below for more information about the Glyco3D resources). A query could also be constructed for specific ligands (e.g., Lewis X). The entries in SugarBindDB are limited to sugar sequences that demonstrate consistent binding to pathogen lectins. Sugar sequences include “glycotope” within a larger carbohydrate structure (the precise lectin-binding site) or entire structures, depending on specific findings within the cited papers. Similar to the GLYCAM-Web server mentioned above, the SugarBindDB is user-friendly in that it allows displaying glycans in a variety of formats; specifically, it supports the modified 2D condensed IUPAC nomenclature, Oxford symbol notation, and the colored CFG cartoons. A sugar query can be input as a structure drawn with GlycanBuilder (18, 19). The SugarBindDB database can also be browsed by disease (e.g., Influenza) or by published references (by author or title descriptions) or searched by the affected area in the pathology (e.g., intestine). Search outputs are very informatively interconnected, i.e., the biological context of each ligand is shown in relation to a disease, to other known ligands, to similar lectins, etc. At the time of writing, over 178 references, 549 pathogenic agents, and 200 ligands have been curated in SugarBindDB.

Glyco3D (25) was developed at the Centre de Recherches sur les Macromolécules Végétales (CERMAV-CNRS), France. It comprises a group of databases containing the 3D data collected from an extensive screening of scientific literature: BiOligo (more than 250 bioactive oligosaccharides along with about 120 of their constituting disaccharide and about 80 monosaccharide segments); PolySac3DB (157 polysaccharides); Lectin3D (more than 1,000 structures); GAG3D (GAG-binding proteins co-crystallized with their ligands); MAbs (a limited set of high resolution structures of carbohydrate–antibody complexes); GT3D (glycosyltransferases crystallized with or without their carbohydrate ligands). Lectins are oligomeric proteins, which can specifically recognize carbohydrates and are involved in many processes crucial to infection and immunity (29); ~70% of the Lectin3D structures are present in complex with a carbohydrate ligand, from monosaccharides to oligosaccharides or glycoproteins, allowing great insight into the molecular basis of carbohydrate recognition by lectins. GAGs are complex anionic polysaccharides (e.g., heparin), specifically recognized by protein receptors, which participate in the regulation of many processes, including cell adhesion. Anti-carbohydrate antibodies, demonstrating specificity to various carbohydrate epitopes, are of a high importance in immunology and vaccine development (30).

The individual databases within Glyco3D are publically accessible via the portal (24), furnished with a user-friendly graphical user interface with several search options. All 3D structures, established using a variety of structure determination methods, can be visualized and some basic measurements are possible on the spot. For a more detailed analysis, the structures can be downloaded in a variety of commonly used molecular file formats.

Why would a user access 3D structural information relevant to glycobiology via databases such as Glyco3D while it can be obtained from the original sources, mainly PDB (31)? The answer is: in databases, such as Glyco3D, the data is manually curated and value-added. Such curation and value-adding is indispensable, given poor quality of structural information for carbohydrates in PDB. Agirre et al. have recently demonstrated that 64% of all *N*-glycan d-pyranosides in PDB reflect poor fit to the electron density (32). They have revealed significant structural errors, such as incorrect conformations, stereochemical configurations, and linkages, resulting from incorrect model building from the start, as well as more subtle errors, resulting from incorrect refinement. Even subtle errors could be detrimental if incorrect structures are interpreted in a biological context. The reasons for errors are various, but the main one seems to be poor chemical understanding on the part of crystallographers and the lack of appropriate torsional restraints, often in disagreement with the low-resolution data. The detriment of such errors goes beyond incorrect interpretations in specific cases. It also leads to skewed statistics based on all published/deposited structures, creating new abnormal “norms.” Thus, structural glycobiology is now ripe for remediation of the type that the protein crystallography field underwent in not too distant past (33).

Tools for structural validation of carbohydrate 3D structural data are available, have been developed specifically for carbohydrates, and are publically accessible (34). For example, pdb-care (35) can check residue notation (which can be a source of confusion for carbohydrate residues) and atom connectivities and evaluate biological correctness of glycan structures. CARP (carbohydrate Ramachandran plot) (36) analyses carbohydrate data by generating a separate Phi/Psi plot for each linkage type, such as in the study of conformational preferences of *Shigella flexneri* O-antigens and their implications for vaccine design (37). Information necessary for such plots can be obtained from the GlyTorsion database (36), containing glycosidic torsions observed in PDB entries, or from conformational maps based on molecular dynamics simulations provided by GlycoMapsDB (38). Both pdb-care and CARP are available at the Glycosciences.de portal (39).

To summarize, the online resources available for structural glycobiology reviewed here demonstrate good scientific citizenship features: multinational collaborative approach to design and development, open access to data and code, and the drive for common and accepted standards. The collaborative approach is illustrated not only by initiatives such as UniCarbKB, but also by efforts to cross-link and integrate existing resources (e.g., SugarBindDB and Glyco3D) and map functional and structural information (e.g., mapping of SugarBindDB to GlycoSuiteDB to link saccharide sequences with their physical location in the body). Further, somewhat altruistic, aspect of these developments is the extensive effort that goes into manual curation of data. Efforts like these should be recognized, credited, and supported more strongly by relevant scientific societies and funding bodies.

## Conflict of Interest Statement

The authors declare that the research was conducted in the absence of any commercial or financial relationships that could be construed as a potential conflict of interest.

## References

[1] von der Lieth C-W Lütteke T Frank M Bioinformatics for Glycobiology and Glycomics: An Introduction. Chichester: John Wiley & Sons (2009). 494 p.

[2] von der Lieth C-W Lütteke T Frank M, editors. Appendix 1: list of available websites. Bioinformatics for Glycobiology and Glycomics. Chichester: John Wiley & Sons (2009). p. 447–52.

[3] Aoki-Kinoshita KF. Using databases and web resources for glycomics research. Mol Cell Proteomics (2013) 12:1036–45. 10.1074/mcp.R112.026252 23325765 PMC3617328

[4] Lutteke T Frank M Glycoinformatics. New York, NY: Springer (2015). 331 p.

[5] GLYCAM Web-Server. (2015). Available from: http://glycam.org/

[6] Complex Carbohydrate Research Center. (2015). Available from: http://www.ccrc.uga.edu/research/index.php

[7] Kirschner KN Yongye AB Tschampel SM González-Outeiriño J Daniels CR Foley BL GLYCAM06: a generalizable biomolecular force field. Carbohydrates. J Comput Chem (2008) 29:622–55. 10.1002/jcc.20820 17849372 PMC4423547

[8] Makeneni S Ji Y Watson DC Young NM Woods RJ. Predicting the origins of anti-blood group antibody specificity: a case study of the ABO A- and B-antigens. Front Immunol (2014) 5:397. 10.3389/fimmu.2014.00397 25202309 PMC4141161

[9] Ramsland P. Blood brothers: carbohydrates in xenotransplantation and cancer immunotherapy. Immunol Cell Biol (2005) 83:315–7. 10.1111/j.1440-1711.2005.01367.x 16033526

[10] Pancera M Zhou T Druz A Georgiev IS Soto C Gorman J Structure and immune recognition of trimeric pre-fusion HIV-1 Env. Nature (2014) 514:455–61. 10.1038/nature13808 25296255 PMC4348022

[11] Campbell MP Peterson R Mariethoz J Gasteiger E Akune Y Aoki-Kinoshita KF UniCarbKB: building a knowledge platform for glycoproteomics. Nucleic Acids Res (2014) 42:D215–21. 10.1093/nar/gkt1128 24234447 PMC3964942

[12] UniCarbKB. (2015). Available from: http://www.unicarbkb.org/

[13] UniCarb-DB. (2015). Available from: http://unicarb-db.biomedicine.gu.se//unicarbdb/show_mucin.action

[14] Cooper CA Harrison MJ Wilkins MR Packer NH. GlycoSuiteDB: a new curated relational database of glycoprotein glycan structures and their biological sources. Nucleic Acids Res (2001) 29:332–5. 10.1093/nar/29.1.332 11125129 PMC29828

[15] GlycoBase. (2015). Available from: https://glycobase.nibrt.ie/glycobase/show_nibrt.action.

[16] Campbell MP Nguyen-Khuong T Hayes CA Flowers SA Alagesan K Kolarich D Validation of the curation pipeline of UniCarb-DB: building a global glycan reference MS/MS repository. Biochim Biophys Acta (2014) 1844:108–16. 10.1016/j.bbapap.2013.04.018 23624262

[17] EUROCarbDB. (2015). Available from: http://www.ebi.ac.uk/pdbe-srv/eurocarbdb/

[18] Damerell D Ceroni A Maass K Ranzinger R Dell A Haslam SM. The GlycanBuilder and GlycoWorkbench glycoinformatics tools: updates and new developments. Biol Chem (2012) 393:1357–62. 10.1515/hsz-2012-0135 23109548

[19] GlycanBuilder. (2015). Available from: http://www.unicarbkb.org/builder

[20] Cooper CA Gasteiger E Packer NH. GlycoMod – a software tool for determining glycosylation compositions from mass spectrometric data. Proteomics (2001) 1:340–9. 10.1002/1615-9861(200102)1:2<340::AID-PROT340>3.0.CO;2-B 11680880

[21] GlycoMod. (2015). Available from: http://web.expasy.org/glycomod/

[22] RINGS. (2015). Available from: http://rings.t.soka.ac.jp/

[23] Functional Glycomics Gateway (CFG). (2015). Available from: http://www.functionalglycomics.org/static/index.shtml

[24] Perez S Sarkar A Breton C Drouillard S Rivet A Imberty A Glyco3D: A Portal for Structural Glycoscience. (2013). Available from: http://glyco3d.cermav.cnrs.fr 10.1007/978-1-4939-2343-4_18 25753716

[25] Pérez S Sarkar A Rivet A Breton C Imberty A Glyco3D: a portal for structural glycosciences. In: Lütteke T Frank M, editors. Glycoinformatics. New York, NY: Springer (2015). p. 241–58. 10.1007/978-1-4939-2343-4_18 25753716

[26] Lee LY Lin CH Fanayan S Packer NH Thaysen-Andersen M. Differential site accessibility mechanistically explains subcellular-specific *N*-glycosylation determinants. Front Immunol (2014) 5:404. 10.3389/fimmu.2014.00404 25202310 PMC4142333

[27] Shakhsheer B Anderson M Khatib K Tadoori L Joshi L Lisacek F SugarBind database (SugarBindDB): a resource of pathogen lectins and corresponding glycan targets. J Mol Recognit (2013) 26:426–31. 10.1002/jmr.2285 23836470

[28] SugarBindDB. (2015). Available from: http://sugarbind.expasy.org/

[29] Sharon N Lis H Lectins. Netherlands: Springer (2007). 454 p.

[30] Dingjan T Spendlove I Durrant LG Scott AM Yuriev E Ramsland PA. Structural biology of antibody recognition of carbohydrate epitopes and potential uses for targeted cancer immunotherapies. Mol Immunol (2015). 10.1016/j.molimm.2015.02.028 25757815

[31] Berman HM Westbrook J Feng Z Gilliland G Bhat TN Weissig H The protein data bank. Nucleic Acids Res (2000) 28:235–42. 10.1093/nar/28.1.235 10592235 PMC102472

[32] Agirre J Davies G Wilson K Cowtan K Carbohydrate anomalies in the PDB. Nat Chem Biol (2015) 11:303 10.1038/nchembio.1798 25885951

[33] Read RJ Adams PD Arendall WB III Brunger AT Emsley P Joosten RP A new generation of crystallographic validation tools for the protein data bank. Structure (2011) 19:1395–412. 10.1016/j.str.2011.08.006 22000512 PMC3195755

[34] Emsley P Brunger AT Lütteke T Tools to assist determination and validation of carbohydrate 3D structure data. In: Lütteke T Frank M, editors. Glycoinformatics. New York, NY: Springer (2015). p. 229–40. 10.1007/978-1-4939-2343-4_17 25753715

[35] Lutteke T von der Lieth CW. pdb-care (PDB carbohydrate residue check): a program to support annotation of complex carbohydrate structures in PDB files. BMC Bioinformatics (2004) 5:69. 10.1186/1471-2105-5-69 15180909 PMC441419

[36] Lütteke T Frank M von der Lieth CW. Carbohydrate structure suite (CSS): analysis of carbohydrate 3D structures derived from the PDB. Nucleic Acids Res (2005) 33:D242–6. 10.1093/nar/gki013 15608187 PMC539967

[37] Theillet FX Simenel C Guerreiro C Phalipon A Mulard LA Delepierre M. Effects of backbone substitutions on the conformational behavior of *Shigella flexneri* O-antigens: implications for vaccine strategy. Glycobiology (2011) 21:109–21. 10.1093/glycob/cwq136 21030536

[38] Frank M Lutteke T von der Lieth CW. GlycoMapsDB: a database of the accessible conformational space of glycosidic linkages. Nucleic Acids Res (2007) 35:287–90. 10.1093/nar/gkl907 17202175 PMC1899098

[39] Glycosciences.de. (2015). Available from: http://www.glycosciences.de/

